# Epiregulin contributes to breast tumorigenesis through regulating matrix metalloproteinase 1 and promoting cell survival

**DOI:** 10.1186/s12943-015-0408-z

**Published:** 2015-07-29

**Authors:** Mariya Farooqui, Laura R. Bohrer, Nicholas J. Brady, Pavlina Chuntova, Sarah E. Kemp, C. Taylor Wardwell, Andrew C. Nelson, Kathryn L. Schwertfeger

**Affiliations:** Department of Lab Medicine and Pathology, University of Minnesota, 2231 6th St SE, Minneapolis, MN 55455 USA; Masonic Cancer Center, University of Minnesota, Minneapolis, MN 55455 USA; Microbiology, Immunology and Cancer Biology Graduate Program, University of Minnesota, Minneapolis, MN 55455 USA; College of Veterinary Medicine, University of Minnesota, Minneapolis, MN 55455 USA

**Keywords:** Breast cancer, Epiregulin, MMP-1, FGFR, Ductal carcinoma *in situ*

## Abstract

**Background:**

The epidermal growth factor (EGF) family of ligands has been implicated in promoting breast cancer initiation, growth and progression. The contributions of EGF family ligands and their receptors to breast cancer are complex, and the specific mechanisms through which different ligands regulate breast tumor initiation and growth are not well-defined. These studies focus on the EGF family member epiregulin (EREG) as a mediator of early stage breast tumorigenesis.

**Methods:**

EREG expression levels were assessed in both cell lines and human samples of ductal carcinoma *in situ* (DCIS) using quantitative RT-PCR, ELISA and immunohistochemistry. Gene knock-down approaches using shRNA-based strategies were used to determine the requirement of EREG for growth of MCF10DCIS cells *in vivo*, and for identifying mechanisms through which EREG promotes tumor cell survival. Experiments were performed using a combination of two-dimensional culture, three-dimensional culture and tumor growth *in vivo*.

**Results:**

In comparison with other EGF family members, EREG was induced in MCF10DCIS cells compared with MCF10A and MCF10AT cells and its expression was partially regulated by fibroblast growth factor receptor (FGFR) activity. Reduced EREG expression in MCF10DCIS cells led to decreased tumor growth *in vivo*, which was associated with reduced cell survival. Furthermore, treatment of MCF10A cells with exogenous EREG enhanced cell survival both in three-dimensional culture and in response to chemotherapeutic agents. Examination of EREG-induced signaling pathways demonstrated that EREG promoted survival of MCF10A cells through regulating expression of matrix metalloproteinase-1 (MMP-1). To determine the relevance of these findings in human tumors, samples of DCIS were analyzed for EREG and MMP-1 expression. EREG was induced in DCIS lesions compared to normal breast epithelium, and EREG and MMP-1 were correlated in a subset of DCIS samples.

**Conclusions:**

Together, these studies lead to identification of a novel pathway involving EREG and MMP-1 that contributes to the formation of early stage breast cancer. Understanding these complex pathways could ultimately lead to the development of novel biomarkers of neoplastic progression and/or new therapeutic strategies for patients with early stage cancer.

**Electronic supplementary material:**

The online version of this article (doi:10.1186/s12943-015-0408-z) contains supplementary material, which is available to authorized users.

## Introduction

The earliest stages of malignant transformation require the acquisition of multiple phenotypes, such as proliferation, survival and migration, which contribute to tumor growth and progression [1]. Identifying the key cellular and molecular factors that drive the formation of early breast cancer lesions will ultimately result in the development of preventive approaches for patients that are at high risk for developing invasive breast cancer. Furthermore, as methods for detecting breast lesions become increasingly sensitive, the successful identification of markers associated with early lesions could lead to better prognostic indicators and/or the development of patient-tailored therapies that inhibit malignant progression [2].

Aberrant activation of the ErbB family of growth factor receptors and their downstream signaling pathways has been implicated in breast cancer initiation and maintenance [3]. Activation of ErbB receptors in cancer occurs through various mechanisms, including mutation, amplification and regulation of epidermal growth factor (EGF)-family ligands and cross-talk with other signaling pathways [3–7]. Several EGF ligands have been implicated in breast cancer, including EGF, epiregulin (EREG), amphiregulin (AREG), heparin-bound EGF (HB-EGF) and transforming growth factor alpha (TGFα) [8–14]. We focus here on EREG, which we demonstrate is increased in ductal carcinoma *in situ* (DCIS) compared with non-transformed breast epithelium. Previous studies found detectable levels of *EREG* mRNA in up to 45.5 % of breast cancer and EREG has been linked to pulmonary metastasis in experimental studies [13, 15, 16]. However, the contributions of EREG to early stages of breast tumor initiation and growth have not been investigated.

We demonstrate here that EREG regulates expression of matrix metalloproteinase-1 (MMP-1) in non-transformed breast epithelial cells and in a model of DCIS. MMP-1 is an interstitial collagenase that has been implicated in breast cancer progression [17, 18]. Expression of MMP-1 was found to be higher in atypical ductal hyperplasia (ADH) from patients that progressed to invasive breast cancer than those from patients that did not progress [19]. Furthermore, high levels of MMP-1 expression are associated with poor prognosis [17] and increased risk of bone metastasis in breast cancer patients [20]. While it is well documented that MMP-1 cleaves extracellular matrix molecules, such as collagen [21, 22], MMP-1 has also been linked to the promotion of cell survival [23, 24], suggesting that MMP-1 may contribute to multiple processes during tumor growth and progression.

In the studies described here, we demonstrate that EREG expression is increased in early stage breast cancer lesions. Furthermore, we use both two-dimensional (2D) and three-dimensional (3D) cell culture assays to demonstrate that EREG acts through induction of MMP-1 to confer survival advantages to non-transformed mammary epithelial cells. Finally, we demonstrate that loss of EREG expression in transformed breast cancer cells leads to reduced tumor growth *in vivo*, which is associated with increased tumor cell apoptosis. Based on these studies, we propose that EREG contributes to the formation of pre-invasive lesions in a subset of breast cancers. Identifying novel mechanisms through which early stage breast cancers arise has important implications for identification of biomarkers of aggressive disease and development of novel therapeutic strategies.

## Results

### Identification of EREG as a potential mediator of early stage breast cancer

**Fig. 1:**
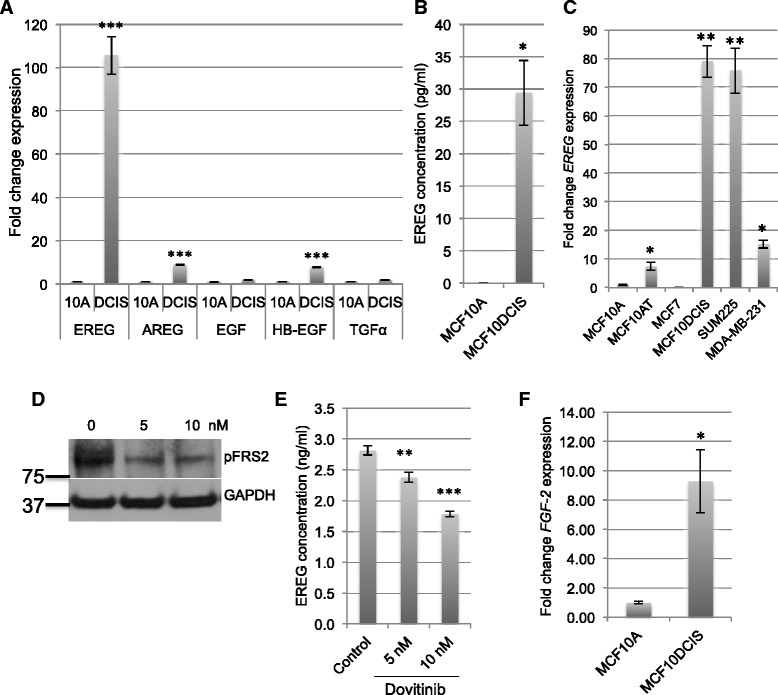
Regulation of EREG expression in MCF10DCIS cells by FGFR activity. **a** qRT-PCR of the indicated EGF ligands was performed on RNA isolated from MCF10A cells and MCF10DCIS cells. Expression levels were normalized to levels of *CYBP*. **b** Soluble levels of EREG in conditioned media obtained from MCF10A and MCF10DCIS cells as determined by ELISA. **c** qRT-PCR of the *EREG* expression was performed on RNA isolated from the indicated cell lines. **d** Immunoblot analysis was performed to examine the effects of the indicated amounts of dovitinib on phosphorylation of FRS-2 in MCF10DCIS cells. **e** Concentration of EREG in conditioned media, as determined by ELISA, from MCF10DCIS cells treated with the indicated amounts of dovitinib for 18 h. **f** qRT-PCR analysis of *FGF-2* expression in MCF10A and MCF10DCIS cells. Levels normalized to *CYBP*. *p < 0.05, **p < 0.001, ***p < 0.0001

EGF ligands and their receptors are well-established mediators of breast cancer growth and progression [3–5, 7]. Lee *et al.* demonstrated that expression levels of both *AREG* and *EREG* were increased in hyperplastic enlarged lobular units compared to normal epithelium isolated from human breast samples, suggesting differential regulation of EGF ligands during the earliest stages of tumor initiation [10]. Thus, an initial screen of EGF ligand expression was performed in MCF10A cells, which represent non-transformed breast epithelial cells, and MCF10DCIS cells, which were derived from MCF10A cells and form tumors that have characteristics of comedo-type DCIS *in vivo* [25]. qRT-PCR was performed to assess expression levels of *EREG*, *AREG*, *EGF*, *HB-EGF* and *TGFα*, all of which have been implicated in breast cancer [8, 10–14]. Expression levels of *EGF* and *TGFα* were not changed between the two cell lines (Fig. 1a). *AREG* and *HB-EGF* were increased approximately 8-fold in the MCF10DCIS cells compared with MCF10A cells (Fig. 1a). However, *EREG* expression levels were found to be increased over 100-fold in MCF10DCIS cells compared with MCF10A cells (Fig. 1a). EREG is expressed as a transmembrane protein and is shed into the media by cell surface proteases [26–28], thus soluble EREG is detectable by ELISA. As shown in Fig. 1b, a significant increase in EREG was found in conditioned media obtained from MCF10DCIS cells compared with media from MCF10A cells.

To assess EREG expression in additional breast cancer cell lines, *EREG* expression levels were examined in additional cell lines including MCF10AT, an HRAS-transformed derivative of the MCF10A cell line, MCF7, an estrogen receptor positive cell line, SUM225, another cell line capable of forming DCIS-like lesions *in vivo* and MDA-MB-231, a triple negative invasive cell line. *EREG* was found to be highest in the MCF10DCIS and SUM225 cells, compared with the other cell lines (Fig. 1c). These findings are consistent with the hypothesis that EREG may be induced in early stages of breast cancer.

In previously published studies, we demonstrated that EGF ligands, including EREG, are regulated by FGFR signaling [29]. To examine whether FGFR activity is linked to the increase in EREG expression in MCF10DCIS cells, cells were treated with the FGFR-selective inhibitor dovitinib. FGFR inhibition led to a decrease in pFRS2, a downstream substrate of FGFR (Fig. 1d), and a significant decrease in EREG expression in a dose dependent manner (Fig. 1e). Notably, the concentration of dovitinib used (5 nM and 10nM) was within the range of specificity for FGFRs [30, 31]. To identify the mechanism through which FGFR is activated in these cells, qRT-PCR analysis was performed to examine expression levels of FGF ligands in serum starved MCF10A and MCF10DCIS cells. Of the 22 ligands examined, two were found to be increased in MCF10DCIS cells more than 2-fold, including *FGF-2*, which was increased 13-fold (Fig. 1f), and *FGF-13*, which was increased 2-fold (data not shown). These results suggest that EREG is regulated, in part, as a result of autocrine FGFR activation in MCF10DCIS cells.

### Reduced expression of EREG in MCF10DCIS cells leads to decreased cell survival

**Fig. 2:**
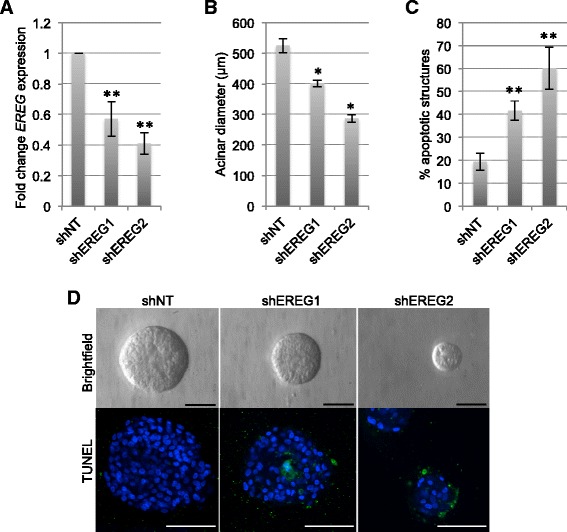
Decreased expression of EREG leads to reduced acinar growth *in vitro*. **a** MCF10DCIS cells were transduced with either non-targeting (NT) or two different EREG shRNA constructs. Expression levels of *EREG* were determined by qRT-PCR and normalized to levels of *CYBP*. **b** Quantification of acinar size. **c** Quantification of apoptotic structures. **d** MCF10DCIS cells expressing either NT shRNA or shEREG were plated in Matrigel and allowed to establish for 4 days followed by treatment with 1 μg/ml doxycycline for an additional 4 days. Upper panels show images obtained by light microscopy. Lower panels show apoptotic cells within structures visualized by TUNEL staining. Scale bars represent 200 μm. *p < 0.01, **p < 0.005

Based on the finding that EREG expression is significantly enhanced in MCF10DCIS compared with MCF10A cells, further studies were performed to determine the contributions of EREG to MCF10DCIS tumor growth. For these studies, EREG gene expression was knocked down in MCF10DCIS cells using a tetracycline-inducible shRNA. Of the four constructs tested, two constructs were chosen for further study based on significant knock-down of *EREG* expression (Fig. 2a). Effects of EREG knock-down on growth of MCF10DCIS cells were initially evaluated in three-dimensional culture. Cells expressing either non-targeting shRNA or an EREG shRNA were plated in Matrigel and allowed to establish for 4 days. The structures were then treated with doxycycline for an additional 6 days and effects on acinar diameter were measured. Loss of EREG led to a decrease in overall acinar size of MCF10DCIS cells (Fig. 2b,d), which correlated with increased apoptosis as determined by TUNEL (Fig. 2c,d).

**Fig. 3:**
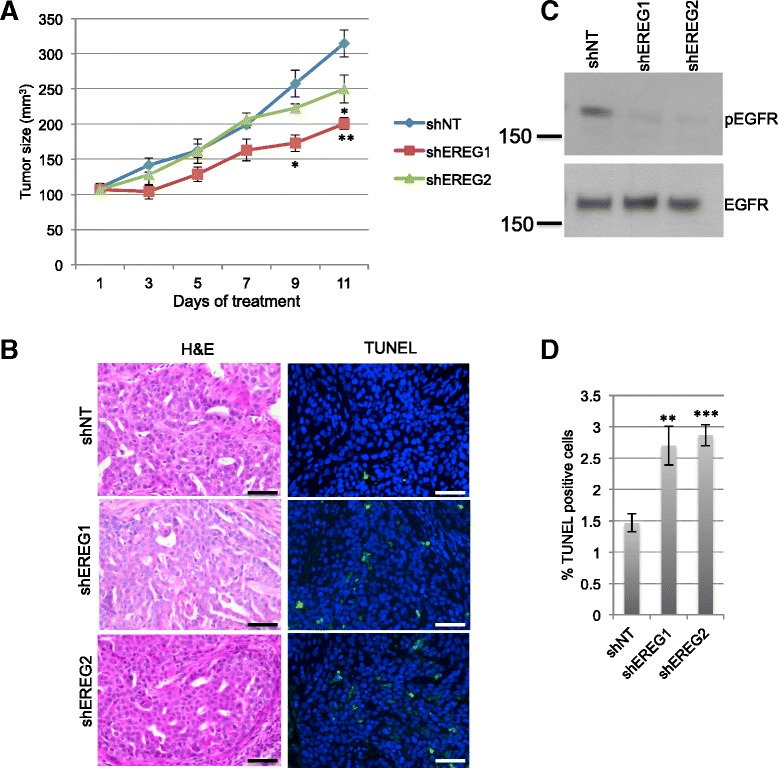
Decreased expression of EREG leads to reduced tumor growth *in vivo*. **a** 50,000 MCF10DCIS-shNT, MCF10DCIS-shEREG1 or MCF10DCIS-shEREG1 cells were injected subcutaneously into nude mice. Once tumors reached 100 mm^3^, mice were provided doxycycline. Tumors were measured every other day. **b** Tumors harvested, sectioned and stained with hematoxylin and eosin (H&E). TUNEL analysis was performed to identify apoptotic cells (*green*). Scale bars represent 50 μm. **c** Immunoblot analysis was performed on protein samples isolated from pooled tumors to detect pEGFR. Immunoblotting of total EGFR was performed to assess equal loading. **d** Quantification of TUNEL-positive cells in tumors from (**a**). *n* = 4 tumors per construct. *p < 0.05, **p < 0.005, ***p < 0.0001

To assess the effects of loss of EREG expression on tumor growth, MCF10DCIS cells expressing either non-targeting shRNA or EREG shRNA were implanted subcutaneously into nude mice. Once the tumors reached a size of 100 mm^3^, the mice were administered doxycycline to induce shRNA expression. As shown in Fig. 3a, reduced EREG expression using both shRNA constructs led to a significant reduction in tumor growth. Despite this reduction in tumor size, the histologic appearance of both control and EREG knockdown tumors was similar (Fig. 3b). Histologic sections from both experimental conditions demonstrated tumor cells growing in tightly packed, solid nests of variable size. Smaller nests exhibited myoepithelial cells consistent with an *in situ* component while larger nests and solid sheets demonstrated direct abutment of tumor cells to desmoplastic stroma consistent with a pushing, invasive border. Comedo-type necrosis with acute inflammation was moderate to widespread, and mitotic figures were abundant. Overall, no single morphologic characteristic could reliably differentiate control from EREG knockdown tumors, and the appearance was highly consistent with previously published reports of the MCF10DCIS xenograft tumors [25]. Despite the morphologic similarity based on H&E staining, immunoblot analysis of tumor lysates demonstrated that loss of EREG was associated with decreased phosphorylation of EGFR (Fig. 3c). While there was no difference in tumor cell proliferation as determined by BrdU incorporation (data not shown), there was a significant increase in apoptosis in the tumors expressing EREG shRNA as determined by TUNEL analysis (Fig. 3b,d). Taken together, these results demonstrate that loss of EREG leads to reduced cell survival *in vivo*.

### EREG promotes cell survival of MCF10A cells in an MMP-1 dependent manner

**Fig. 4:**
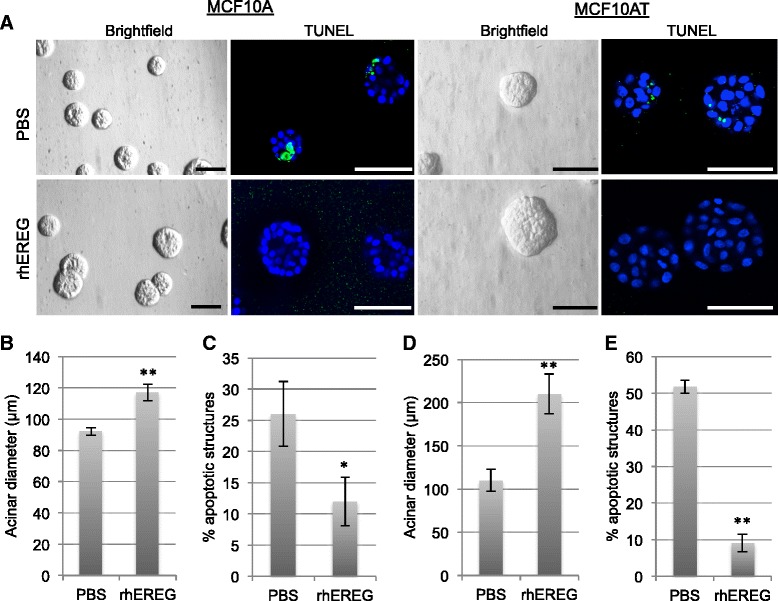
EREG promotes survival of MCF10A cells in 3D culture and in response to chemotherapeutic agents. **a** MCF10A and MCF10AT cells were plated in recombinant basement membrane in the presence of either 10 ng/ml rhEREG or solvent control (PBS). Light microscopy was used to examine effects of EREG on acinar morphology. Structures were stained for cleaved caspase-3 (*green*) and DAPI (*blue*) and analyzed by confocal microscopy. Scale bars represent 100 μm (MCF10A) and 200 μm (MCF10AT). **b** Quantification of acinar diameter of MCF10A structures. **c** Quantification of MCF10A structures with at least one cleaved caspase-3 positive cell. **d** Quantification of acinar diameter of MCF10AT structures. **e** Quantification of MCF10AT structures with at least one cleaved caspase-3 positive cell. *p < 0.05, **p < 0.001

In addition to knock-down experiments in MCF10DCIS cells, the consequences of enhancing EREG-mediated signaling in both MCF10A and MCF10AT cells were examined. Studies were performed in 3D culture to examine effects of EREG on epithelial morphogenesis. When plated in recombinant basement membrane matrix, MCF10A cells undergo both proliferation and apoptosis to form acinar-like structures [32]. MCF10A and MCF10AT cells were plated in recombinant basement membrane and allowed to grow for 8 days in the presence of either rhEREG or PBS as a solvent control. There was a moderate but significant increase in acinar size when MCF10A cells were grown in the presence of rhEREG (Fig. 4a,b). Further analysis of proliferation and apoptosis in these structures revealed that there was no increase in proliferation as determined by phospho-histone H3 staining (data not shown). However, there was a significant decrease in apoptosis as determined by staining structures for cleaved caspase-3 (Fig. 4a,c). As an additional control, similar studies were performed using the MCF10AT cells, which were transformed using an activated HRAS [33] and also express relatively low levels of EREG (Fig. 1c). Treatment of MCF10AT cells with EREG led to an increase in size of acinar structures that was more robust than the induction in size of the MCF10A structures (Fig. 4a,d). Similarly, we found a reduction in the percentage of apoptotic structures upon treatment with rhEREG (Fig. 4a,e).

**Fig. 5:**
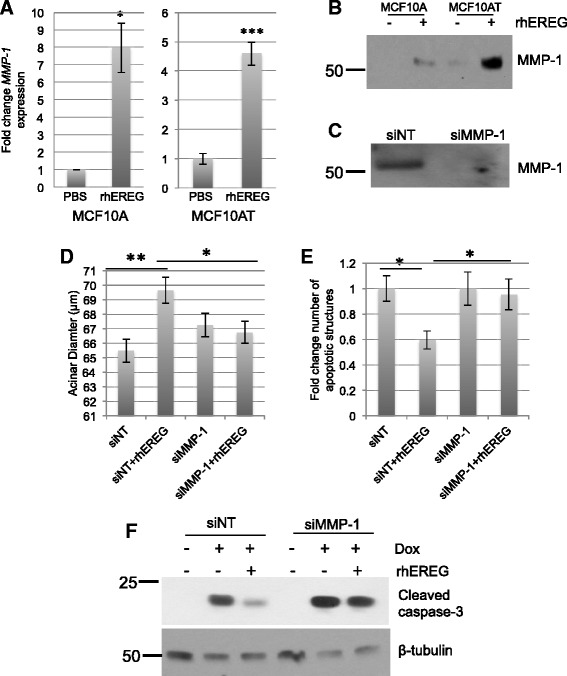
MMP-1 contributes to EREG-induced survival of MCF10A cells. **a** qRT-PCR analysis of *MMP-1* gene expression in MCF10A and MCF10AT cells treated with either solvent (PBS) or 10 ng/ml rhEREG. Expression levels of *MMP-1* were normalized to levels of *CYBP*. **b** Immunoblot analysis of MMP-1 protein in conditioned media obtained from MCF10A and MCF10AT cells following 24 h of rhEREG treatment. Loading was assessed by Coomassie staining (Additional file 1: Figure S1A). **c** Immunoblot analysis of MMP-1 protein in conditioned media obtained from MCF10A cells treated with either non-targeting (NT) or MMP-1 siRNA following 24 h of rhEREG treatment. Loading was assessed by Coomassie staining (Additional file 1: Figure S1B). **d** Quantification of acinar diameter of structures. MCF10A cells were transfected with either NT or MMP-1 siRNA prior to plating in recombinant basement membrane with either solvent (PBS) or 10 ng/ml rhEREG. **e** Quantification of apoptotic structures. Structures that were positive for at least one cleaved caspase-3 positive cell were counted. **f** MCF10A cells were treated with non-targeting (NT) or MMP-1 siRNA, serum starved and treated with solvent control (PBS) or with 10 ng/ml rhEREG in the presence or absence of 2 μM doxorubicin (Dox). Effects on apoptosis were assessed by immunoblot analysis for cleaved caspase-3. β-tubulin is shown as a loading control. *p < 0.05, **p < 0.001, ***p < 0.0001

To identify potential mechanisms through which EREG contributes to survival of MCF10A cells, a candidate gene approach with a focus on target genes known to be associated with early stage breast lesions was taken. MMP-1 was identified as a candidate gene of interest based on published studies demonstrating that MMP-1 expression is enhanced in pre-invasive lesions (ADH) that progress to invasive breast cancers. Although MMP activity is frequently attributed to invasion, MMP-1 has also been linked to cell survival [23, 24], thus representing a reasonable candidate gene for further analysis. Treatment of MCF10A and MCF10AT cells with rhEREG led to increased expression of MMP-1 gene expression and protein expression, with a stronger induction in MMP-1 protein observed in the MCF10AT cells (Fig. 5a,b). To assess the contributions of MMP-1 to EREG-mediated cell survival, MMP-1 expression was knocked down using siRNA strategies (Fig. 5c). Knockdown of MMP-1 in MCF10A cells abrogated the ability of rhEREG treatment to enhance acinar size (Fig. 5d) and prevent apoptosis as evidenced by increased levels of caspase-3 cleavage in 3D culture (quantified in Fig. 5e). In addition to examining the effects of EREG on morphogenesis, further studies were performed to examine the effects of rhEREG on cell survival in response to an apoptotic stimulus. MCF10A cells were treated with the chemotherapeutic agent doxorubicin in the presence and absence of rhEREG. While doxorubicin induced apoptosis, as shown by enhanced expression of cleaved caspase-3, the addition of rhEREG to the media promoted resistance to doxorubicin-induced apoptosis (Fig. 5f). Furthermore, MMP-1 knockdown in MCF10A cells abrogated the ability of rhEREG treatment to prevent doxorubicin-induced apoptosis as evidenced by increased levels of caspase-3 cleavage (Fig. 5f). These studies suggest that the EREG-MMP-1 pathway promotes survival of MCF10A cells.

### Correlation between EREG and MMP-1 expression in human breast cancers

**Fig. 6:**
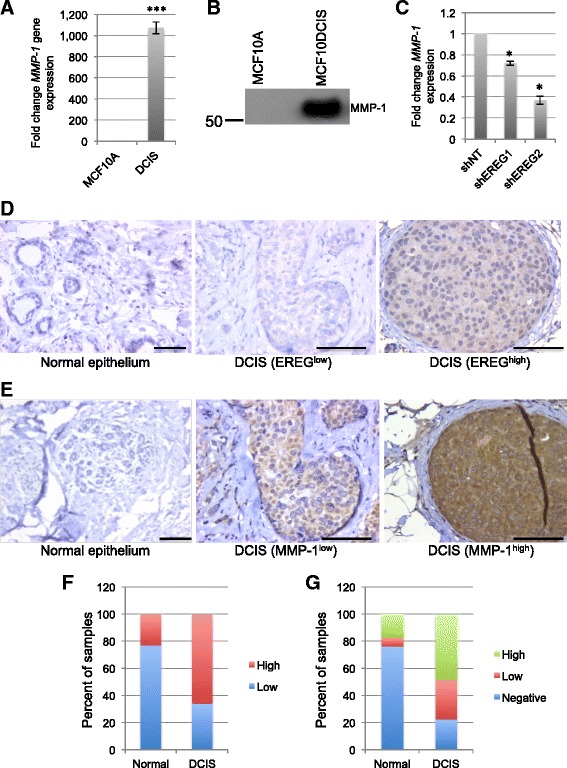
EREG expression correlates with MMP-1 levels in human DCIS lesions. **a** qRT-PCR analysis of *MMP-1* gene expression in MCF10A and MCF10DCIS cells. Expression levels of *MMP-1* are normalized to *CYBP*. **b** Immunoblot analysis of MMP-1 in conditioned media obtained from MCF10A and MCF10DCIS cells. Loading was assessed by Coomassie staining (Additional file 1: Figure S1C). **c** qRT-PCR analysis of *MMP-1* gene expression in MCF10DCIS cells expressing either NT or EREG shRNA constructs. Expression levels of *MMP-1* are normalized to *CYBP*. **d** Immunohistochemistry of normal breast tissue and DCIS stained with an anti-EREG antibody. Scale bars represent 50 μm. **e** Normal and DCIS human samples were stained with an anti-MMP-1 antibody. Representative images of normal and varying levels of MMP-1 staining in DCIS lesions are shown. **f** Quantification of the percent of samples staining positive for EREG. **g** Quantification of the percent of samples staining positive for MMP-1. Scale bars represent 50 μm. *p < 0.05. ***p < 0.0001

To determine whether loss of EREG expression correlated with altered MMP-1 expression in MCF10DCIS cells, MMP-1 expression was examined. Increased gene and protein expression of MMP-1 was found in MCF10DCIS cells compared with MCF10A cells (Fig. 6a,b). Analysis of *MMP-1* expression in MCF10DCIS cells with reduced EREG expression demonstrated a decrease in *MMP-1* expression (Fig. 6c). These results suggest that similar to MCF10A cells, EREG also regulates expression of MMP-1 in MCF10DCIS cells.

Based on our findings in the MCF10A and MCF10DCIS cells, we analyzed expression levels of both EREG and MMP-1 in human DCIS lesions. Normal (*n* = 17) and DCIS lesions (*n* = 31) were stained for EREG expression. While 29.4 % of the normal samples showed expression of EREG, 64.5 % of DCIS lesions expressed EREG (*p* < 0.05) (Fig. 6d,f). Together, these results suggest that EREG expression is relatively low in non-transformed breast epithelial cells, but that its expression increases in a percentage of DCIS lesions. To determine whether EREG and MMP-1 are coordinately expressed in human tumors, the same normal and DCIS human samples were stained with an antibody to MMP-1 and expression levels were scored. We found that high levels of MMP-1 were found in 48 % of samples and low/negative levels were found in 52 % of samples (Fig. 6e,g). Furthermore, there was a significant correlation between EREG and MMP-1 expression (p = 0.011) with EREG being expressed in 70 % of the MMP-1^high^ tumors (Table 1). Thus, EREG and MMP-1 expression are positively correlated in a subset of DCIS tumors.

**Table 1:** MMP-1 expression by EREG expression

	Low EREG	High EREG
No MMP-1	5 (45.5 %)	2 (10.0 %)
Low MMP-1	5 (45.5 %)	4 (20.0 %)
High MMP-1	1 (9.0 %)	14 (70.0 %)

*p*-value for the association between EREG and MMP-1 expression = 0.011

## Discussion

While there have been numerous studies focusing on the consequences of EGFR activation in breast cancer [4, 5, 7], less is known regarding the specific mechanisms through which the different EGF ligands promote early breast cancer growth and progression. Based on findings in which EREG was found to be highly upregulated in MCF10DCIS cells in comparison with MCF10A cells, the studies described here focus on determining the potential contributions of EREG to early stages of breast tumor formation. Expression profiling studies have suggested that both *EREG* and *AREG* expression levels are enhanced in early stage breast lesions compared with normal epithelium [10]. Furthermore, EREG expression has been suggested to be a marker of early stages of other types of cancers, such as ovarian cancer [34]. Finally, mislocalization of the EREG transmembrane protein, which is typically expressed at the basolateral cell surface, to the apical cell surface enhances transformation of epithelial cells [28]. Thus, understanding how EREG might contribute to early stages of tumorigenesis has the potential to lead to novel biomarkers of early stage disease or to the development of preventive or therapeutic approaches.

Based on the finding that EREG was enhanced in MCF10DCIS cells, initial studies were performed to identify mechanisms driving enhanced EREG expression. Our work has focused primarily on identifying important FGFR-induced target genes that contribute to pro-tumorigenic phenotypes. In previous studies, we demonstrated that FGFR activation leads to increased expression and production of EGF ligands, including AREG and EREG, which act through EGFR to enhance FGFR-mediated signaling [29]. Published microarray studies of early breast lesions, specifically hyperplastic enlarged lobular units, identified both *AREG* and *EREG* as genes that were significantly induced in these lesions [10]. Here we demonstrate that FGFR activation in MCF10DCIS cells, possibly through increased production of FGF ligands such as FGF-2, contributes at least partially to the increase in EREG observed in these cells. Previously published studies have demonstrated increased production of FGF-2 as a mechanism for enhanced FGFR activity in triple negative breast cancers [35]. Thus it is possible that similar mechanisms are involved in early stage cancer as well.

We demonstrate that EREG signaling promotes cell survival, which contributes to increased tumor volume *in vivo*. While it is possible that the reduction in tumor volume solely reflects an overall decrease in EGF ligand concentration within the tumor, it is also possible that EREG has distinct effects on tumor growth than the other ligands. Treatment of non-transformed cells with exogenous EREG reduced apoptosis in 3D culture, a process that is critical for epithelial morphogenesis in this model [36]. In addition, EREG reduced sensitivity of cells to chemotherapy. Together, these studies suggest that EREG functions primarily to promote cell survival in these cells. Using a candidate gene approach to identify potential EREG regulated genes that are associated with both cell survival and pre-invasive lesions, we found MMP-1 to be induced following EREG stimulation of MCF10A cells. The studies described here link MMP-1 directly to EREG-induced mammary epithelial cell survival both in response to apoptosis-inducing agents in 2D culture and during epithelial morphogenesis in 3D culture. These results suggest that MMP-1 expression may be important for promoting survival of epithelial cells during tumor initiation. Furthermore, high levels of MMP-1 expression in tumors may confer resistance to chemotherapeutic treatments. Further studies are warranted to define the specific mechanisms of MMP-1 action during these distinct processes.

The results from these studies suggest that early stage tumor cells produce increased levels of EREG, and that exogenous EREG is capable of enhancing survival of epithelial cells even in the presence of serum and additional EGF ligand in the context of 3D culture. This observation raises the possibility that increased levels of EREG production by tumor cells could potentially affect neighboring non-transformed epithelial cells. EREG was found to significantly enhance survival of non-transformed epithelial cells, leading to a possible model in which high levels of EREG in the developing tumor microenvironment may enhance tumorigenic properties of surrounding normal epithelium in a paracrine manner.

Analysis of *EREG* and *MMP-1* expression in the TCGA database did not reveal a significant association, possibly due to the fact that these samples represent invasive breast cancers, rather than early stage breast cancers. However, expression of EGFR and MMP-1 demonstrate a significant trend towards co-occurrence (*p* < 0.001), suggesting that this pathway may be maintained in invasive breast cancers.

## Conclusions

In summary, our studies have identified a novel pathway that promotes early stages of tumorigenesis. While further studies are required to fully implicate this pathway in promoting the development of early stage lesions, they provide a foundation with which to better understand the complex interactions involved in promoting tumorigenesis.

## Materials and methods

### Cell culture

MCF10A cells were obtained from the American Type Culture Collection (ATCC) and were maintained in DMEM/F12 (Lonza, Allendale, NJ) supplemented with 5 % horse serum (Life Technologies, Carlsbad, CA), 20 ng/ml EGF (Life Technologies), 0.5 μg/ml hydrocortisone (Sigma, St. Louis, MO), 100 ng/ml cholera toxin (Sigma), 10 μg/ml insulin (Akron Biotech, Boca Raton, FL), and 1 % penicillin-streptomycin (Life Technologies). MCF10AT, MCF10DCIS and SUM225 cells were obtained from Dr. Fariba Behbod (University of Kansas Medical Center). MCF10AT cells were maintained in the same media as used for the MCF10A cells. MCF10DCIS cells were maintained in DMEM/F12, 5 % horse serum, and antibiotic/antimycotic (Life Technologies). 293 T cells were obtained from ATCC and maintained in D-MEM supplemented with 10 % FBS and 1 % penicillin-streptomycin (Life Technologies). MCF7 and MDA-MB-231 cells were obtained from ATCC and maintained as recommended. For treatment of cells with EREG, cells were treated with 10 ng/ml recombinant human EREG (rhEREG) (1195-EP-025/CF; R&D Systems, Minneapolis, MN). For inhibitor studies, cells were treated with the indicated amounts of dovitinib (TKI-258, LC Laboratories) or the appropriate amounts of solvent control.

### Immunoblot analysis

For signaling analysis, cells were lysed in radioimmunoprecipitation assay (RIPA) buffer and 20 μg of protein were analyzed using SDS-PAGE. The following antibodies were used for immunoblotting: phospho-FRS2α (3861), GAPDH (2118), β-tubulin (2146) pEGFR (2234) and cleaved caspase 3 (9661) (Cell Signaling, Danvers, MA). To analyze MMP-1 by immunoblot analysis, conditioned medium was collected from MCF10A and MCF10DCIS cells and MMP-1 expression was determined (MAB901, R&D Systems). Equal amounts of conditioned media were analyzed and loading was examined by staining the gels with Coomassie and full gel images are included in Additional file 1: Figure S1.

### Epiregulin ELISA

Equal numbers of MCF10A and MCF10DCIS cells were plated in growth media for 24 hours and serum starved for an additional 24 h. The media was replaced with serum free media and the concentration of EREG in the conditioned media was determined 24 h later using the Human EREG ELISA according to the manufacturer’s protocol (Biosource).

### Quantitative reverse transcription PCR(qRT-PCR)

RNA was extracted from cells using Trizol (Life Technologies) as described in the manufacturer’s protocol. cDNA was made using the iScript cDNA synthesis kit (Bio-Rad, Hercules, CA) as described in the manufacturer’s protocol. Quantitative RT-PCR was performed using iQ SYBR green supermix (Bio-Rad) and the Bio-Rad iQ5 system. The 2^-ΔΔCt^ method [37] was used to determine the relative quantification of gene expression, which was normalized to *cyclophilin B (CYBP)*. Primer sequences are listed in Additional file 2: Table S1.

### Three-dimensional culture assay

2500 MCF10A cells or MCF10AT cells were plated on growth factor reduced Matrigel (BD Biosciences, San Jose, CA) as described [32] and treated with PBS or 10 ng/ml rhEREG on the day of plating (day 0). Images were taken at 20X and acinar diameter was calculated on day 8. For detecting apoptosis, cultures were fixed with 2 % paraformaldehyde on day 5 and stained with cleaved caspase-3 (9661; Cell Signaling) as described [32] and mounted with ProLong Gold with DAPI (Life Technologies). MCF10DCIS cells expressing non-targeting shRNA or shRNAs specific for EREG were plated into Matrigel in the same media used for the MCF10A cells [32] and allowed to establish for 4 days. Structures were treated with 1 μg/ml doxycycline for an additional 6 days prior to imaging, fixing and staining as described above. Confocal images were taken at the confocal microscopy facility at the University of Minnesota Masonic Cancer Center.

### Gene knockdown

MMP-1 was transiently knocked down in MCF10A cells using siRNA approaches. Human MMP-1 and non-targeting (NT) ON-TARGETplus SMART-pool siRNAs (Dharmacon, Lafayette, CO) were used according to manufacturer’s protocols. Knockdown was confirmed by immunoblot analysis using conditioned media. For detecting apoptosis in the cells with MMP-1 knockdown, cells were treated with siRNA for 24 h and then plated in Matrigel and analyzed as described above. Stable knock-down of EREG in MCF10DCIS cells was obtained by retroviral transduction of cells with EREG shRNA or non-targeting (NT) tetracycline-inducible TRIPZ vectors (Open Biosystems, Pittsburgh, PA). Briefly, 293 T cells were transfected with the indicated constructs using the Trans-Lentiviral Packaging System (Open Biosystems, Pittsburgh, PA). MCF10DCIS cells were transduced with the virus-containing media and stable cells were selected using 2.5 μg/ml puromycin. shRNA expression was induced by doxycycline (1 μg/ml) as indicated.

### Tumor studies

FOXN1Nu athymic nude mice (Harlan Laboratories, Indianapolis, IN) were injected subcutaneously with MCF10DCIS expressing non-targeting or EREG shRNA (50,000 cells in 50 μl of 50 % Matrigel [38]). Subcutaneous injections were performed rather than orthotopic transplantation due to the need for consistent detection of early tumors (100 mm^3^) prior to shRNA induction by doxycycline. Mice were palpated and measured using calipers. Once tumors reached 100 mm^3^, mice were given 2 g/kg doxycycline in the chow (Harlan Laboratories) to induce EREG knockdown. After 11 days, mice were sacrificed and tumors were fixed in 4 % paraformaldehyde for 2 h embedded in paraffin and cut to 5 μm sections for histological analysis by staining with hematoxylin and eosin. Four tumors were analyzed per shRNA construct. All animal care and procedures were approved by the Institutional Animal Care and Use Committee of the University of Minnesota and were in accordance with the procedures detailed in the Guide for Care and Use of Laboratory Animals.

### Immunohistochemistry and immunofluorescence

Antigen retrieval was performed on tissue sections using a sodium citrate based buffer (Vector Laboratories, Burlingame, CA). Tissues were blocked with 5 % BSA/0.5 % Tween and incubated with primary antibodies overnight. For immunohistochemistry with MMP-1 (MAB901; R&D Systems) and EREG (AF1195; R&D Systems) and biotinylated anti-mouse or anti-goat secondary antibody (Vector Laboratories) were used following heat based antigen retrieval (Vector Laboratories). Antibody was detected using a Vectastain ABC kit (Vector Laboratories), visualized with DAB (Vector Laboratories) and counterstaining was performed with hematoxylin. For immunofluorescence, sections were stained using the TUNEL kit (Promega, Madison, WI) according to the manufacturer’s protocol.

### Tissue Microarrays (TMAs)

TMAs, which were approved by the UMN Institutional Review Board, were obtained from the UMN BioNet Core Facility as described previously [39]. Areas of DCIS were verified by a pathologist and coded specimens and data were provided for this study. All patient identification was redacted and not available to authors per BioNet IRB approval. A total of 17 normal and 31 DCIS samples were analyzed for EREG and MMP-1 expression. Due to technical issues including loss of sample and different core areas, combined analysis included 11 normal and 20 DCIS samples.

### Statistics

Statistical analysis was performed using the unpaired student’s *t*-test to compare two means. Error bars represent the standard error of the mean. For the human samples, the association between EREG and MMP-1 was evaluated using the Chi-squared test.

## Additional files

Additional file 1: Figure S1. Coomassie stained gels for loading controls. A) Loading control for Fig. 5b. B) Loading control for Fig. 5d. C) Loading control for Fig. 6b. Additional file 2: Table S1. Primer sequences used for quantitative RT-PCR.
