# Supplementary figures and images for: Epiregulin contributes to breast tumorigenesis through regulating matrix metalloproteinase 1 and promoting cell survival
Source: Mol Cancer. 2015 Jul 29;14:138. doi: 10.1186/s12943-015-0408-z (PMC4517352; doi:10.1186/s12943-015-0408-z)

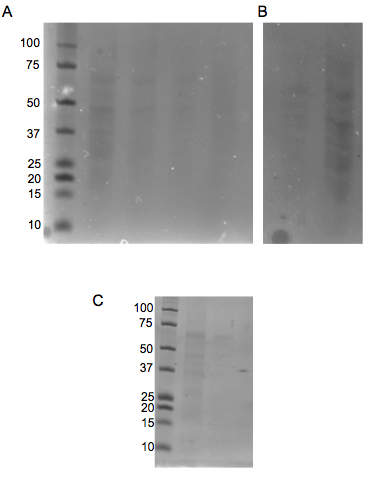

Supplement: Additional file 1: Figure S1. — Coomassie stained gels for loading controls. A) Loading control for Fig. 5b. B) Loading control for Fig. 5d. C) Loading control for Fig. 6b. [file 12943_2015_408_MOESM1_ESM.tiff]
