# Supplementary material for: Epiregulin contributes to breast tumorigenesis through regulating matrix metalloproteinase 1 and promoting cell survival
Source: Mol Cancer. 2015 Jul 29;14:138. doi: 10.1186/s12943-015-0408-z (PMC4517352; doi:10.1186/s12943-015-0408-z)
Supplement: Additional file 2: Table S1. — Primer sequences used for quantitative RT-PCR. [file 12943_2015_408_MOESM2_ESM.docx]

| **Gene** | **Forward Primer** | **Reverse Primer** |
| --- | --- | --- |
| AREG | 5'-GTGGTGCTGTCGCTCTTGATA-3' | 5'-ACTCACAGGGGAAATCTCACT-3' |
| Cyclophillin B | 5'-GAAAGAGCATCTACGGTGAGC-3' | 5'-GTCTTGACTGTCGTG ATGAAGAA-3' |
| EGF | 5'-TGTCCACGCAATGTGTCTGAA-3' | 5'-CATTATCGGGTGAGGAACAACC-3' |
| EREG | 5'-CTGCCTGGGTTTCCATCTTCT-3' | 5'-GCCATTCATGTCAGAGCTACACT-3' |
| HB-EGF | 5'-ATCGTGGGGCTTCTCATGTTT-3' | 5’-TTAGTCATGCCCAACTTCACTTT-3' |
| MMP-1 | 5'-CTGGCCACAACTGCCAAATG-3' | 5'-CTGTCCCTGACCAGCCCAGTACTTA-3' |
| TGFα | 5'-AGGTCCGAAAACACTGTGAGT-3' | 5’-AGCAAGCGGTTCTTCCCTTC-3' |
| FGF-2 | 5'-AAG CGG CTG TAC TGC AAA AAC-3' | 5'-TGA GGG TCG CTC TTC TCC C-3' |

**Supplemental Table 1: Primer sequences used for quantitative RT-PCR.**
